# Cell Death Induction by MO2455, a Novel Potential Anti-Cancer Agent in Monocytic and T-Cell Leukemia Cells

**DOI:** 10.3390/cells15191780

**Published:** 2026-09-30

**Authors:** Ankitha Vadi Velu, Kenji Matsuno, Barkha Saraswat, Osamu Ohno, Yu Yingchun, Zhao Zhenkai, Ying Tong, Takahiro Tougan, Taiki Aoshi, Takeji Takamura, Fumiaki Koizumi, Mitsuko Masutani

**Affiliations:** 1 Department of Molecular and Genomic Biomedicine, Center for Bioinformatics and Molecular Medicine, Graduate School of Biomedical Sciences, Nagasaki University, Nagasaki 852-8523, Japan; bb55322801@ms.nagasaki-u.ac.jp (A.V.V.); jj20260079@ms.nagasaki-u.ac.jp (B.S.); y-tong@nagasaki-u.ac.jp (Y.T.);; 2 Department of Pharmacy, Yasuda Women’s University, Hiroshima 731-0153, Japan; 3 Department of Chemistry and Life Science, School of Advanced Engineering, Kogakuin University, Tokyo 192-0015, Japan; ohno@cc.kogakuin.ac.jp; 4 Department of Immunology, Graduate School of Biomedical Sciences, Nagasaki University, Nagasaki 852-8523, Japan; bb55425004@ms.nagasaki-u.ac.jp (Y.Y.); bb55425003@ms.nagasaki-u.ac.jp (Z.Z.); ttougan@nagasaki-u.ac.jp (T.T.); aoshi@nagasaki-u.ac.jp (T.A.); 5 Faculty of Engineering, Kanagawa Institute of Technology, Atsugi 243-0292, Japan; takamura@chem.kanagawa-it.ac.jp

**Keywords:** leukemia, apoptosis, DNA damage, cell death, mitochondrial dysfunction, imatinib derivative

## Abstract

Despite advances in targeted therapies, treatment resistance and disease recurrence remain major obstacles in leukemia management. Structural modification of clinically successful kinase inhibitors represents a promising approach for discovering novel anti-leukemic agents with improved biological activities. In the present study, we investigated the anti-leukemic activity and mechanism of action of MO2455, a newly synthesized imatinib-derived compound, in monocytic leukemia U937, THP−1, and T-cell leukemia CCRF-CEM cells. MO2455 showed substantial cytotoxic activity against all three cell lines, with IC_50_ values of 0.83 μM, 0.43 μM, and 0.57 μM in U937, THP−1, and CCRF-CEM cells, respectively. Treatment with MO2455 induced early mitochondrial membrane depolarization, internucleosomal DNA laddering, a significant accumulation of sub−G1 population and increased Annexin V positivity, suggesting typical apoptosis without cell cycle arrest in U937 cells. MO2455 induced γ-H2AX induction and upregulated the pro-apoptotic *NOXA* and *BBC3* genes. THP−1 and CCRF-CEM cells displayed distinct large DNA fragmentation with smearing. In PBMCs from two healthy donors, IC_50_ values were 0.99 and 0.60 μM, respectively, although the cells remained morphologically healthy without obvious extensive cell death. Overall, MO2455 exhibits substantial anti-leukemic activity and induces cell death through apoptosis and other mechanisms, supporting further investigation as an anti-leukemic agent.

## 1. Introduction

Leukemia represents a diverse group of hematological malignancies that continue to pose a significant clinical burden worldwide [1,2,3,4]. Despite the availability of chemotherapy, targeted therapies, and hematopoietic stem cell transplantation, many patients experience treatment failure due to resistance or disease recurrence [5,6,7,8]. These limitations highlight the need for the continued development of novel anti-leukemic compounds capable of effectively inducing malignant cell death while overcoming existing therapeutic challenges [9,10].

The introduction of imatinib demonstrated that targeting tyrosine kinase activity can be an effective therapeutic strategy for the treatment of leukemia and has contributed significantly to the development of targeted cancer therapies [11,12,13]. Imatinib targets BCR-ABL, KIT, and platelet-derived growth factor receptor (PDGFR) kinases and has demonstrated remarkable clinical efficacy in several malignancies [14,15]. However, acquired resistance and variable therapeutic responses have prompted continued efforts to develop structurally modified derivatives with improved anti-cancer activity [16]. Many effective anti-leukemic agents induce DNA damage and activate apoptotic pathways, particularly those involving mitochondrial dysfunction and BCL-2 family-regulated signaling [17,18,19]. Nevertheless, the anti-leukemic potential and molecular mechanisms of newly synthesized imatinib-derived compounds remain incompletely characterized [20,21].

In the present study, we evaluated the anti-leukemic activity of MO2455, a novel imatinib-derived compound, in monocytic leukemia U937, THP−1, and T-cell leukemia CCRF-CEM cells. These cell lines provide complementary models for assessing anti-leukemic activity across distinct hematological backgrounds. We investigated whether MO2455 induces cytotoxicity through DNA damage-associated apoptotic signaling and examined the key molecular events involved in this process.

We hypothesized that MO2455 promotes leukemic cell death by activating the intrinsic apoptotic pathway. To test this hypothesis, we assessed cell viability, DNA fragmentation, mitochondrial membrane potential, cell cycle distribution, apoptosis, expression of pro-apoptotic genes, and γ-H2AX accumulation following MO2455 treatment. Our findings demonstrate that MO2455 exerts potent cytotoxic effects on leukemia cells and provides mechanistic evidence of apoptosis and necrosis-like cell death signaling pathways.

## 2. Materials and Methods

### 2.1. Cell Culture

U937, THP−1, and CCRF-CEM cells were obtained from the Japanese Collection of Research Bioresources (JCRB) Cell Bank. For the culture medium, RPMI1640 medium (GIBCO, Life Technologies Corp., Grand Island, NY, USA) with 10% fetal bovine serum (FBS, HyClone Laboratories, Inc., Logan, UT, USA) and 1% penicillin-streptomycin (GIBCO) was used at 37 °C under 5% CO_2_.

### 2.2. Isolation of Human Peripheral Blood Mononuclear Cells (PBMCs)

Peripheral blood mononuclear cells (PBMCs) were isolated from peripheral blood obtained from two healthy donors with informed consent and designated as PBMC-1 and PBMC-2, respectively. This study was conducted with the approval of the Genetic and Medical Ethics Commission at Nagasaki University (protocol code no. 25053003). Briefly, 5 mL of whole blood from each donor was collected into heparinized tubes and gently mixed by inversion. Ficoll-Paque™ PLUS (Cytiva, Uppsala, Sweden; cat. No. 17144002) was used for density-gradient separation. Blood was diluted with sterile phosphate-buffered saline (PBS) and carefully layered onto Ficoll-Paque PLUS in 12 mL Leucosep™ polypropylene tubes (Greiner Bio-One, Frickenhausen, Germany; cat. No. 163290). The samples were centrifuged at 252× *g* for 30 min at 25 °C using a swing-out rotor (Kubota Shoji Co., Ltd., Tokyo, Japan) without disturbing the gradient. Following centrifugation, the whitish interphase containing PBMCs was carefully collected. The isolated cells were transferred to a fresh centrifuge tube and washed twice with sterile PBS by centrifugation at 252× *g* for 10 min. After the final wash, the cell pellet was resuspended in RPMI medium and the cell number and viability were determined.

### 2.3. Chemicals

Dimethyl sulfoxide (DMSO; Fujifilm Wako Pure Chemical Corporation, Osaka, Japan; cat. no. 043-07211); MO2455 (5-[[ (2,2′-Bithiophen)-5-ylcarbonyl]amino]-*N*-[5-[[4-[4-[(4-ethyl-1-piperazinyl)-carbonyl]phenyl]-2-pyrimidinyl]amino]-2-methylphenyl]-3-pyridinecarboxamide) was synthesized in-house and purified to 97.48% as described in Supplementary Materials [22]. The physical appearance of MO2455 is a white solid. A 5 mM stock solution of MO2455 was prepared in 100% DMSO and stored at −80 °C until use. Working solutions were freshly prepared by diluting the stock solution with the appropriate cell culture medium immediately before each experiment. The final concentration of DMSO in all treatment and control groups was maintained at 0.1% (*v*/*v*). The concentrations used for mechanistic studies (1, 2, and 4 μM) were selected based on the cytotoxicity determined after 72 h of treatment.

### 2.4. Cytotoxicity Assay

The cytotoxicity of MO2455 was measured using a Cell Counting Kit-8 (CCK-8) assay kit (Dojindo Laboratories, Mashiki, Kumamoto, Japan). U937, THP−1, and CCRF-CEM cells were seeded at 4000 cells/well, while PBMCs were seeded at 2 × 10^4^ cells/well in 100 μL of medium in 96-well plates, followed by treatment with MO2455. A higher cell density was used for PBMCs than for the cancer cell lines because PBMCs are non-proliferative or slowly proliferating under the experimental conditions and generally require a higher initial cell number to obtain a reliable CCK-8 signal. The CCK assay was carried out as per the manufacturer’s instructions on day 3, with absorbance measurement at 450/620 nm, was measured using an Infinite F50 microplate reader (Tecan Austria GmbH, Grödig, Austria), and the half-maximal inhibitory concentration (IC_50_) value was calculated using a localized linear interpolation model flanking the 50% viability threshold.

### 2.5. Apoptosis Assay

A total of 5 × 10^5^ cells/well were seeded in 2 mL of medium in 6-well plates and treated with the indicated concentrations of MO2455. Cells were subsequently harvested, washed with cold PBS, resuspended in binding buffer, and processed using the FITC Annexin V Apoptosis Detection Kit with PI (BioLegend, San Diego, CA, USA) and then immediately analyzed by the FACSVerse system (BD Biosciences, Mountain View, CA, USA), and data were processed with FlowJo software FlowJo™ v10.10.0 software (BD Life Sciences, Ashland, OR, USA).

### 2.6. Cell Cycle Analysis

For cell cycle analysis, cells were seeded at 5 × 10^5^ cells/well in 2 mL of medium in 6-well plates and treated with the indicated concentrations of MO2455. The cells were then fixed in 70% ethanol, washed with PBS containing 3% (*v*/*v*) FBS, and treated with 0.2 mg/mL RNase A (Sigma-Aldrich; St. Louis, MO, USA; cat. no. R4642) at 37 °C for 60 min. Subsequently, cells were stained with 2 mg/mL propidium iodide (PI) (FUJIFILM Wako Pure Chemical Corporation, Osaka, Japan; cat. no. 163-26284) at 4 °C for 30 min and analyzed with the FACSVerse system and the data were processed with the Watson Pragmatic Model of FlowJo software.

### 2.7. Western Blot Analysis

For Western blot analysis, 1.9 × 10^6^ cells/well were seeded in 2 mL of medium in 6-well plates and treated with the indicated concentrations of MO2455. The cells were harvested, washed with cold PBS, and lysed in Laemmli’s buffer. After SDS-PAGE, the proteins were transferred to a polyvinylidene difluoride (PVDF) membrane (Bio-Rad Laboratories, Inc., Hercules, CA, USA) and treated with primary and corresponding secondary antibodies then detected using Immobilon Western Chemiluminescent HRP Substrate (Millipore, Burlington, MA, USA). The following antibodies were utilized in this study: Rabbit anti-phospho-Histone H2A.X (Ser139) (20E3) monoclonal antibody (Cell Signaling Technology, Inc., Danvers, MA, USA; cat. no. 9718; dilution, 1:1000) and mouse anti-β-actin monoclonal antibody (Sigma-Aldrich; Merck KGaA, Darmstadt, Germany; cat. no. A2228; dilution, 1:10,000).

### 2.8. Analysis of Mitochondrial Membrane Potential

Cells were seeded at 1 × 10^5^ cells/well in 2 mL of medium in 6-well plates and treated with the indicated concentrations of MO2455. The change in mitochondrial membrane potential was measured using the fluorescent dye JC-1 from the JC-1 MitoMP Detection Kit (Dojindo Molecular Technologies, Rockville, MD, USA). The cells were stained with 2 μM JC-1 and incubated in the dark at 37 °C for 30 min. Flow cytometry analysis was performed using the FACSVerse system, and the data were analyzed using FlowJo software.

### 2.9. RNA Isolation and RT-PCR Analysis of mRNA Expression

Cells were seeded at 4 × 10^5^ cells/well in 2 mL of medium in 6-well plates and treated with the indicated concentrations of MO2455. The cells were harvested after 2.5, 5, and 10 h. Total RNA was isolated using RNA lysis buffer (Nippon Gene Co., Ltd.). RT-PCR was performed using *BBC3*, *NOXA*, and *BAX*-specific primers, with *GUSB* as the internal control, using SYBR Select master mix (Applied Biosystems, Thermo Fisher Scientific, Baltics UAB, Vilnius, Lithuania; cat. no. 4472908) in an Applied Biosystems StepOnePlus Real-Time PCR System (Thermo Fisher Scientific, Waltham, MA, USA). The primer sequences (5′-3′, forward and reverse) were as follows: *NOXA*, AAGAACGCTCAACCGAGCCC & GAAGGAGTCCCCTCATGCAAGT; *BBC3*, GGACGACCTCAACGCACAG & ATTGGGCTCCATCTCGGGGG; *BAX*, AACATGGAGCTGCAGAGGATGATT & TCTGATCAGTTCCGGCACCTT; *GUSB*, GCCTGCGTCCCACCTAGAAT & ACATACGGAGCCCCCTTGTC.

### 2.10. DNA Fragmentation Assay

For the DNA fragmentation assay, cells were seeded at 4 × 10^5^ cells/well in 2 mL of medium in 6-well plates and treated with the indicated concentrations of MO2455. Further, the cells were harvested, washed with PBS, and lysed in a buffer containing 10 mM Tris–Cl (pH 7.4), 10 mM EDTA (Sigma-Aldrich, St. Louis, MO, USA), and 0.5% Triton X-100 (Nacalai Tesque, Inc., Kyoto, Japan) and treated with proteinase K (Zymo Research Corp., Irvine, CA, USA; cat. no. D3001-2-F) and RNase A (Sigma-Aldrich; Merck KGaA, Darmstadt, Germany; cat. no. R4642), followed by genomic DNA purification and ethanol precipitation. DNA was loaded onto a 2% agarose gel alongside a 1 kb DNA ladder (New England Biolabs, Inc., Ipswich, MA, USA) and electrophoresed in 1xTAE buffer, stained with ethidium bromide (Thermo Fisher Scientific, Waltham, MA, USA) at 0.5 mg/mL, and visualized under UV illumination on a Gel Doc XR+ Molecular Imager (Bio-Rad Laboratories, Inc., Hercules, CA, USA).

### 2.11. Statistical Analysis

All data are presented as mean ± SE values. Statistical analyses were performed using JMP^®^ Student Edition 19.1.0 (JMP Statistical Discovery LLC, Cary, NC, USA). The Tukey–Kramer HSD test was used to examine the data in this study. The values * *p* < 0.05, ** *p* < 0.001, *** *p* < 0.001, and **** *p* < 0.0001 vs. control were considered to indicate statistical significance. Statistical analyses were performed on experiments with three independent biological replicates.

## 3. Results

### 3.1. MO2455 Induces Mitochondrial Dysfunction and Apoptosis in U937 Cells

**Figure 1:**
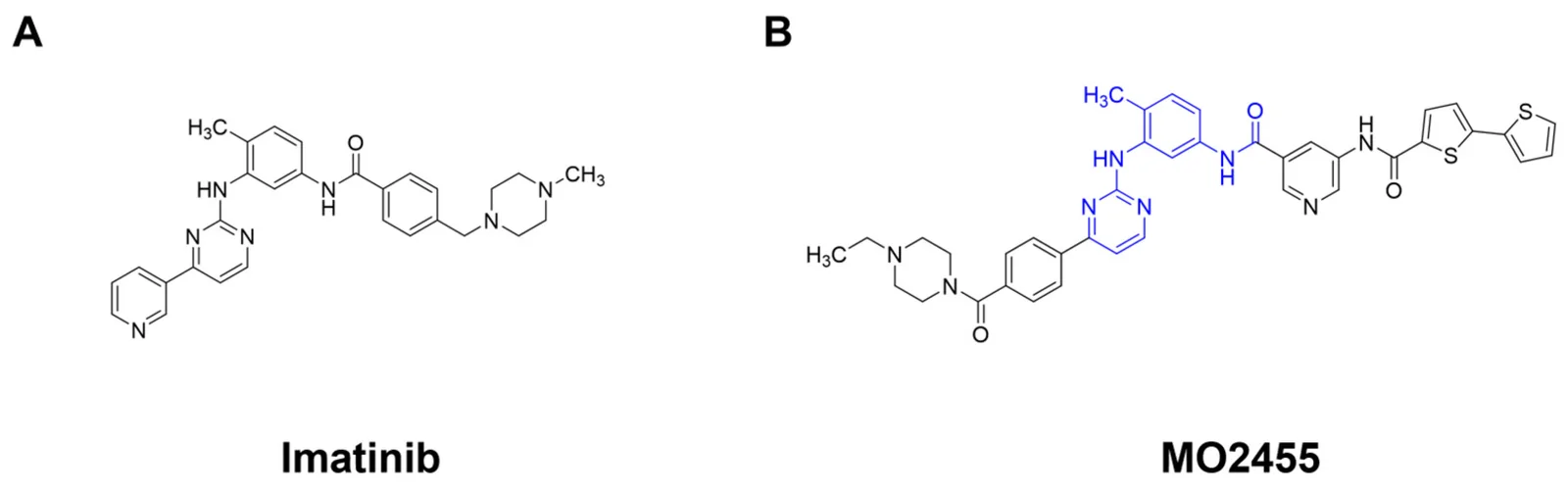
Chemical structures of imatinib (**A**) and MO2455 (**B**). The blue part in MO2455 shows the common structure with imatinib.

In this study, we used MO2455, a novel compound developed from the parental compound imatinib. As shown in Figure 1, MO2455 and imatinib differ in chemical structure by a large percentage, although MO2455 retains the fragmental trait of imatinib. Regarding lipophilicity, the cLogP value for imatinib is 4.38 and that for MO2455 is 7.53, indicating a higher lipophilicity for MO2455. Here in this study, we did not intend to compare the biological effect of MO2455 and imatinib and we analyzed the biological effect as a new class of chemicals. The chemical structures of imatinib and MO2455 are shown in Figure 1A,B.

#### 3.1.1. Cytotoxicity and Genomic DNA Fragmentation Induced by MO2455 in U937 Cells

To investigate the anti-leukemic activity of MO2455, U937 cells were treated with MO2455 and evaluated for changes in cell viability, apoptosis, mitochondrial function, DNA damage and apoptotic gene expression.

**Figure 2:**
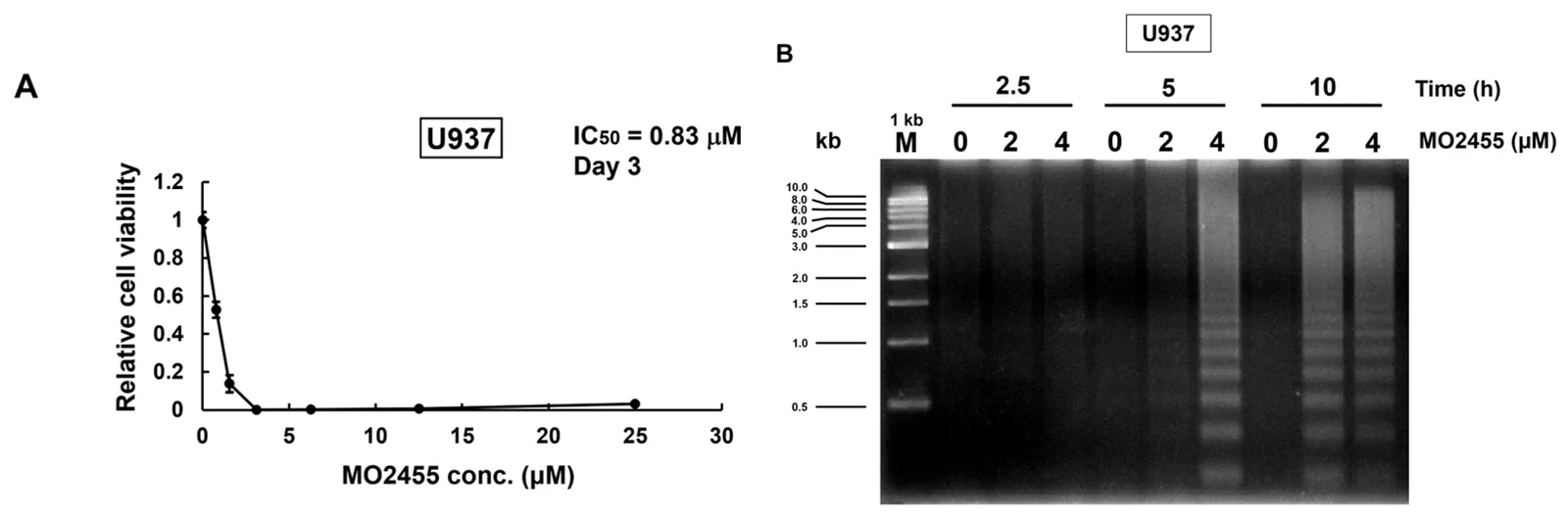
Cytotoxicity and genomic DNA fragmentation induced by MO2455 in U937 cells (**A**) Cytotoxicity of U937 cells following treatment with MO2455 was assessed using the CCK-8 assay on day 3. Cells were treated with different concentrations of MO2455 for an exposure time of 3 days (72 h). Data are presented as the mean ± SE derived from a representative experiment performed in technical triplicate (n = 3). The IC_50_ was calculated. (**B**) Genomic DNA fragmentation was analyzed by agarose gel electrophoresis. Cells were treated with 0, 2, and 4 μM MO2455 for exposure times of 2.5, 5, and 10 h. Data from three independent experiments (n = 3) show reproducible patterns.

CCK-8 assay analysis revealed a substantial reduction in viability following MO2455 treatment for 3 days against U937 cells with an IC_50_ value of 0.83 μM (Figure 2A). To determine whether the reduction in viability was associated with apoptotic cell death, DNA fragmentation was evaluated. After 5 h, faint internucleosomal DNA laddering was evident in cells treated with 2 μM MO2455, whereas a more pronounced laddering pattern was observed at 4 μM. After 10 h, clear apoptotic DNA fragmentation was observed at both concentrations (Figure 2B).

#### 3.1.2. Effects of MO2455 on Mitochondrial Membrane Potential, Cell Cycle Distribution, and Apoptotic Cell Death in U937 Cells

**Figure 3:**
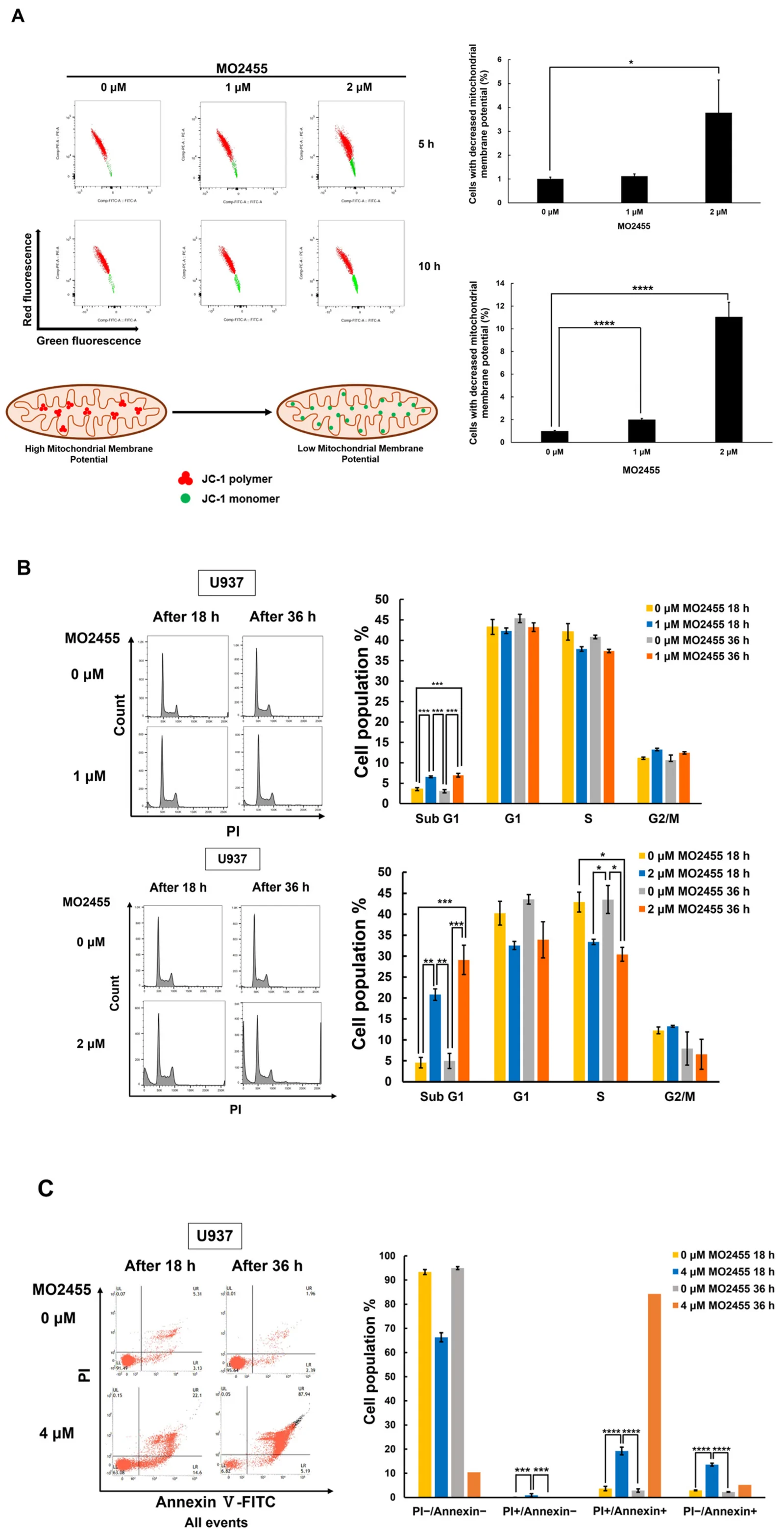
Effects of MO2455 on mitochondrial membrane potential, cell cycle distribution, and apoptosis in U937 cells. (**A**) The changes in the mitochondrial membrane potential of U937 cells following treatment with MO2455. Cells were treated with 0, 1, and 2 μM MO2455 for exposure times of 5 h, and 10 h. (**B**) Flow cytometry analysis after MO2455 treatment. Cells were treated with MO2455 at concentrations of 0, 1, and 2 μM for 18 h or 36 h. After PI staining, cell cycle phases were analyzed. (**C**) Cells were treated with MO2455 at concentrations of 0, and 4 μM for 18 h or 36 h and the rate of apoptosis was evaluated using the FITC Annexin V Apoptosis Detection Kit with PI. Where applicable, data are presented as mean ± SE. Data from three independent experiments (n = 3). For the 4 μM, 36 h sample, (n = 2) conditions were used. Data represents two independent experiments (n = 2) due to limited viable cell recovery following treatment with 4 μM MO2455 for 36 h. Comparisons of all pairs using Tukey–Kramer HSD were carried out. * *p* < 0.05, ** *p* < 0.001, *** *p* < 0.001, and **** *p* < 0.0001 vs. control.

The mitochondrial membrane potential (ΔΨm) was measured using JC-1 staining. Treatment with 2 μM MO2455 induced a reduction in ΔΨm at 5 h. At 10 h, both 1 µM and 2 µM concentrations caused a significant decrease in ΔΨm compared with the untreated controls (Figure 3A) (*p* < 0.0001), indicating early disruption of mitochondrial integrity.

Next, DNA content and cell cycle analyses were performed using PI staining and flow cytometry. MO2455 treatment resulted in a significant accumulation of cells in the sub−G1 phase. Increased sub−G1 populations were observed at both 1 µM and 2 µM after 18 h of treatment and became more pronounced at 36 h, showing a dose- and time-dependent manner (Figure 3B). On the other hand, although the sub−G1 population increased, the percentages of G1, S and G2/M phases decreased after MO2455 treatment and no particular cell cycle arrest was induced, suggesting MO2455 did not cause direct DNA damage. Treatment with 1 μM MO2455 resulted in a significant increase in the sub−G1 population of U937 cells compared with the untreated control (*p* < 0.001). Although a basal sub−G1 population was present in untreated cells, the observed increase following MO2455 treatment indicates enhanced DNA fragmentation associated with apoptotic cell death.

The mode of cell death was further characterized using Annexin V-FITC/PI staining. Treatment with 4 μM MO2455 significantly increased both Annexin+/PI− and Annexin+/PI+ populations at 18 and 36 h relative to untreated controls (Figure 3C), indicating a rapid progression from early apoptosis to late apoptosis and then secondary necrosis with prolonged exposure.

#### 3.1.3. Effects of MO2455 on the Expression of Pro-Apoptotic Genes and γ-H2AX Protein in U937 Cells

**Figure 4:**
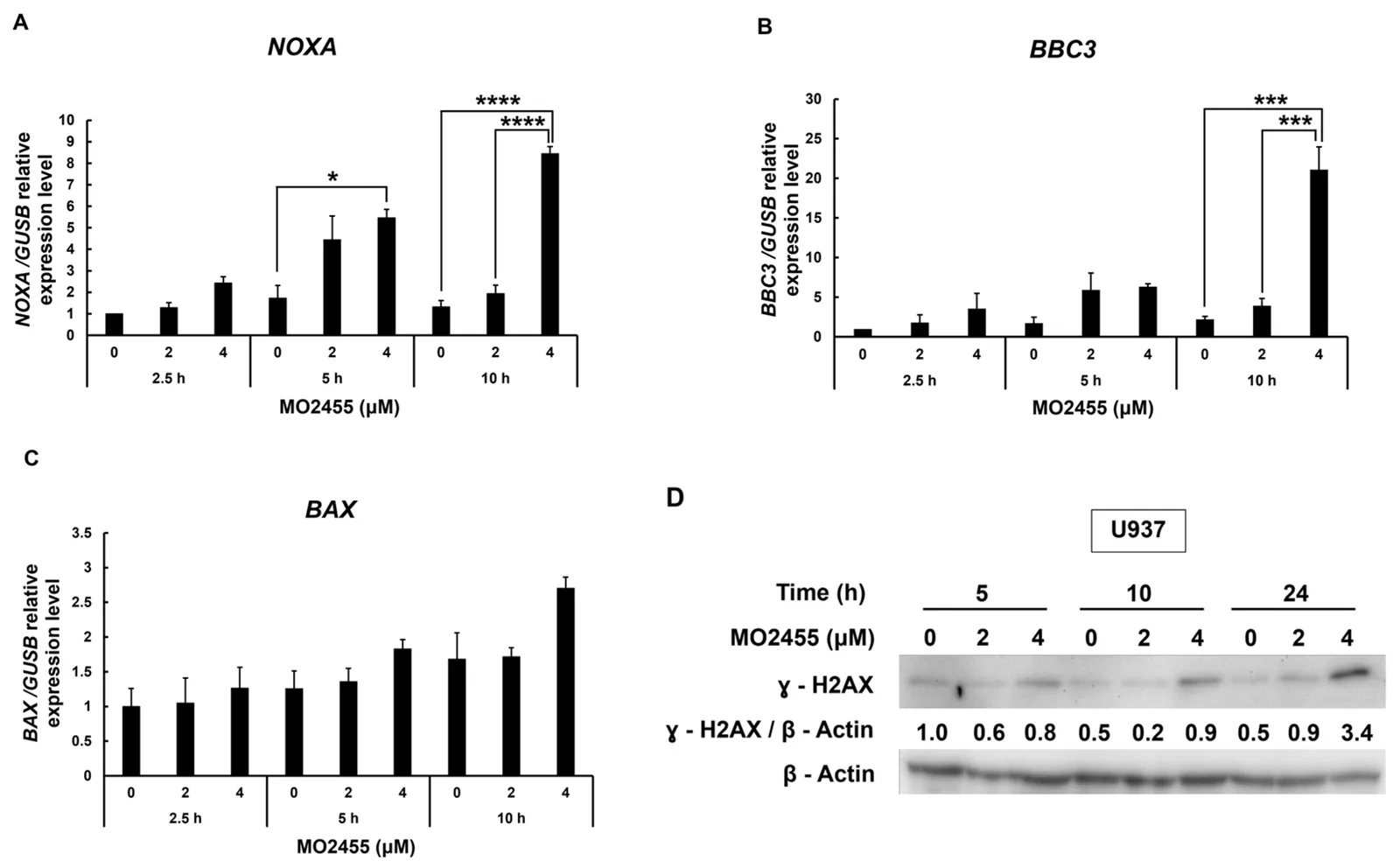
Effects of MO2455 on the expression of pro-apoptotic genes and γ-H2AX protein in U937 cells. (**A**–**C**) The expression levels of pro-apoptotic genes *NOXA* (**A**), *BBC3* (**B**), and *BAX* (**C**) in U937 cells following treatment with MO2455 at concentrations of 0, 2, and 4 μM for 2.5, 5, and 10 h. Gene expression levels were normalized to the housekeeping gene *GUSB*. For panels (**A**,**B**), the 0 μM, 2.5 h group sample, (n = 2) conditions were used. Data represents two independent experiments (n = 2). (**D**) γ-H2AX protein level detected by Western blot analysis. β-Actin was used as a loading control and the ratio of γ-H2AX/β-actin is shown below the blot of γ-H2AX. Where applicable, data are presented as mean ± SE. Data from three independent experiments (n = 3). Comparisons of all pairs using Tukey–Kramer HSD were carried out. * *p* < 0.05, *** *p* < 0.001, and **** *p* < 0.0001 vs. control.

To investigate the molecular events associated with apoptosis, the expression of key pro-apoptotic genes was examined. *NOXA*, *BBC3* (*PUMA*), and *BAX* genes are known to be induced during apoptosis, which is regulated by p53 pathway activation [23,24,25]. As shown in Figure 4A, *NOXA* expression was significantly induced from 5 h and reached a higher level at 10 h. *BBC3* expression also increased and reached statistical significance at 10 h (Figure 4B). *BAX* expression showed an upward trend after treatment (Figure 4C). The timing of *NOXA* and *BBC3* was paralleled with the decrease in ΔΨm and DNA ladder formation. Because *NOXA* and *BBC3* are frequently induced by genotoxic stress [26,27], DNA damage was evaluated by examining γ-H2AX levels. Western blot analysis showed increased γ-H2AX levels in cells treated with 4 μM MO2455, with expression increasing over time and reaching the highest level at 24 h (Figure 4D). It has been reported that increased γ-H2AX levels are associated with apoptosis-induced DNA fragmentation [28,29]. Because the increase in γ-H2AX levels was paralleled with the timing of apoptosis induction, it can be suggested that apoptotic DNA fragmentation, such as DNA double-strand breaks, caused the increase in γ-H2AX levels. Collectively, the results demonstrate that MO2455 exerts potent cytotoxic effects in U937 cells through apoptosis induction associated with mitochondrial depolarization, pro-apoptotic gene expression, DNA fragmentation, sub−G1 accumulation, and Annexin V positivity, which is consistent with rapid activation of the intrinsic apoptotic pathway.

### 3.2. MO2455 Promotes Necrotic Cell Death in THP−1 Cells

**Figure 5:**
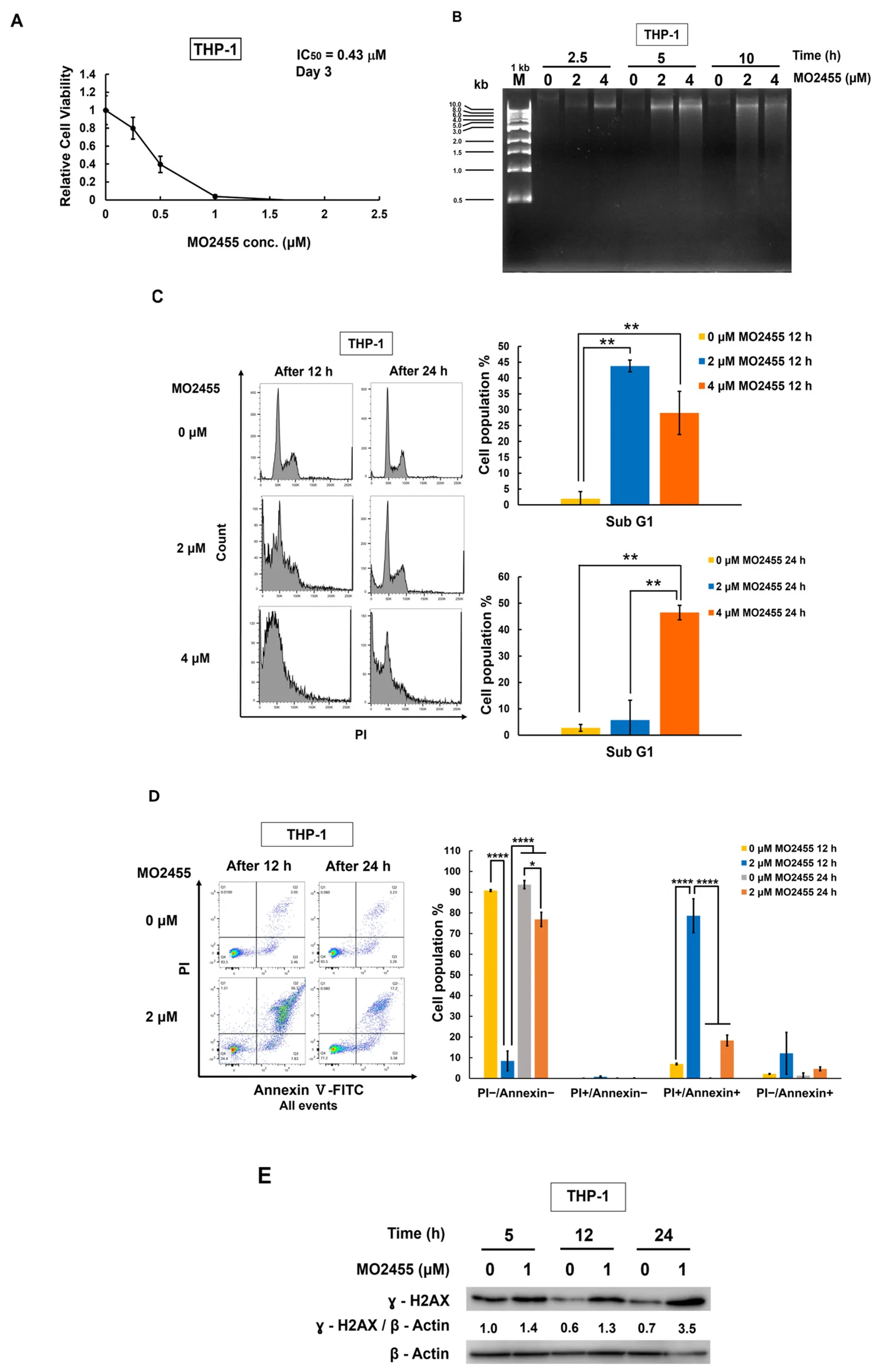
Effect of MO2455 on THP −1 cells. (**A**) Cytotoxicity assessed using the CCK assay on day 3. Cells were treated with different concentrations of MO2455 for an exposure time of 3 days (72 h). Data are presented as the mean ± SE. derived from a representative experiment performed in technical triplicate (n = 3). The IC_50_ value was then calculated. (**B**) Genomic DNA fragmentation analysis in THP −1 cells. Cells were treated with 0, 2, and 4 μM MO2455 for exposure times of 2.5, 5, and 10 h. Data from three independent experiments (n = 3) show reproducible patterns. (**C**) Flow cytometry analysis after MO2455 treatment in THP−1 cells. Cells were treated with MO2455 at concentrations of 0, 2, and 4 μM for 12 h or 24 h. After PI staining, sub−G1 population levels were analyzed. Other cell cycle phases could not be calculated because of extensive DNA fragmentation after MO2455 treatment. (**D**) Cells were treated with MO2455 at concentrations of 0, and 2 μM for 12 h or 24 h and the rate of apoptosis was evaluated using the FITC Annexin V Apoptosis Detection Kit with PI. (**E**) γ-H2AX protein level was detected by Western blot analysis. β-Actin was used as a loading control and the ratio of γ-H2AX/β-actin is shown below the blot of γ-H2AX. Data are presented as mean ± SE. Data from three independent experiments (n = 3). Comparisons of all pairs using Tukey–Kramer HSD were carried out. * *p* < 0.05, ** *p* < 0.001 and **** *p* < 0.0001 vs. control.

To evaluate the anti-leukemic activity of MO2455 in other monocytic leukemia cells, cell viability was assessed in THP−1 cells using the CCK assay. Treatment with MO2455 resulted in a dose-dependent reduction in cell viability, with an IC_50_ value of 0.43 μM, indicating potent cytotoxic activity (Figure 5A). DNA fragmentation analysis showed that, in contrast to the characteristic internucleosomal DNA laddering observed in U937 cells, THP−1 cells exhibited larger DNA fragmentation at >10 kb with smearing from 2.5 h. Treatment with 2 μM and 4 μM MO2455 for 5 and 10 h produced pronounced high molecular weight (>10 kb) DNA fragmentation with smearing, further increased by 2 and 4 μM MO2455 treatment (Figure 5B); however, a typical apoptotic DNA ladder formation was not observed. The effect of MO2455 on DNA content and cell cycle was examined using flow cytometry analysis of PI-stained cells. MO2455 induced a marked increase in the sub−G1 population at 12 h. Elevated sub−G1 fractions were observed at both 12 and 24 h, with the highest accumulation detected in the 2 and 4 μM treated cells at 12 and 24 h, respectively (Figure 5C). Due to the extensive increase in sub−G1 fractions, the calculation of G1, S, and G2/M populations using FlowJo software was not possible. The extensive increase in the sub−G1 fractions induced by MO2455 treatment was consistent with large DNA fragmentation and cell death.

To further characterize the mode of cell death, Annexin V-FITC/PI staining was performed in THP−1 cells. MO2455 treatment significantly increased the Annexin+/PI+ population compared to that in the untreated controls at both 12 h and 24 h, corresponding to late apoptotic/necrotic cells (Figure 5D). The marked increase in the double-positive population indicates rapid progression to cell death, accompanied by the loss of membrane integrity. In the assay with FITC Annexin V Apoptosis Detection Kit with PI, unfixed cells were gated using the FSC/SSC gating procedure. Therefore, the sub−G1 fragmented and smaller, secondary necrotic cells could be detected as a PI+/Annexin+ population in apparently amplified numbers. As shown in Figure 5C, 2 μM MO2455 treatment in THP−1 cells induced a marked increase in the sub−G1 fragmented (or smaller-sized) population to around 40% at 12 h and reflecting this, the PI+/Annexin+ population in unfixed cells became around 60% as apparently amplified numbers as shown in Figure 5D. At 24 h after 2 μM MO2455 treatment, as shown in Figure 5C, the sub−G1 population decreased to around 25%, possibly through further fragmentation and less recovery during centrifugation procedures during the preparation. Consistent with this change, the PI-/Annexin-population percentage in unfixed cells at 24 h became apparently higher than that at 12 h as shown in Figure 5D, right panel.

To investigate DNA damage, γ-H2AX expression was analyzed using Western blotting. Increased γ-H2AX levels were detected at all examined time points (5, 12, and 24 h) following MO2455 treatment (Figure 5E), indicating activation of the DNA damage response. The sustained elevation of γ-H2AX could be associated with DNA double-strand break formation due to the extensive DNA fragmentation during cell death, as observed in U937 cells after MO2455 treatment. These results demonstrate that MO2455 exhibits potent cytotoxic effects in THP−1 cells and induces cell death associated with larger-sized DNA fragmentation, suggesting a mixed cell death with substantial necrotic or parthanatos-like features following MO2455 treatment.

### 3.3. MO2455 Causes Cytotoxicity and Induces Apoptosis in CCRF-CEM Cells

**Figure 6:**
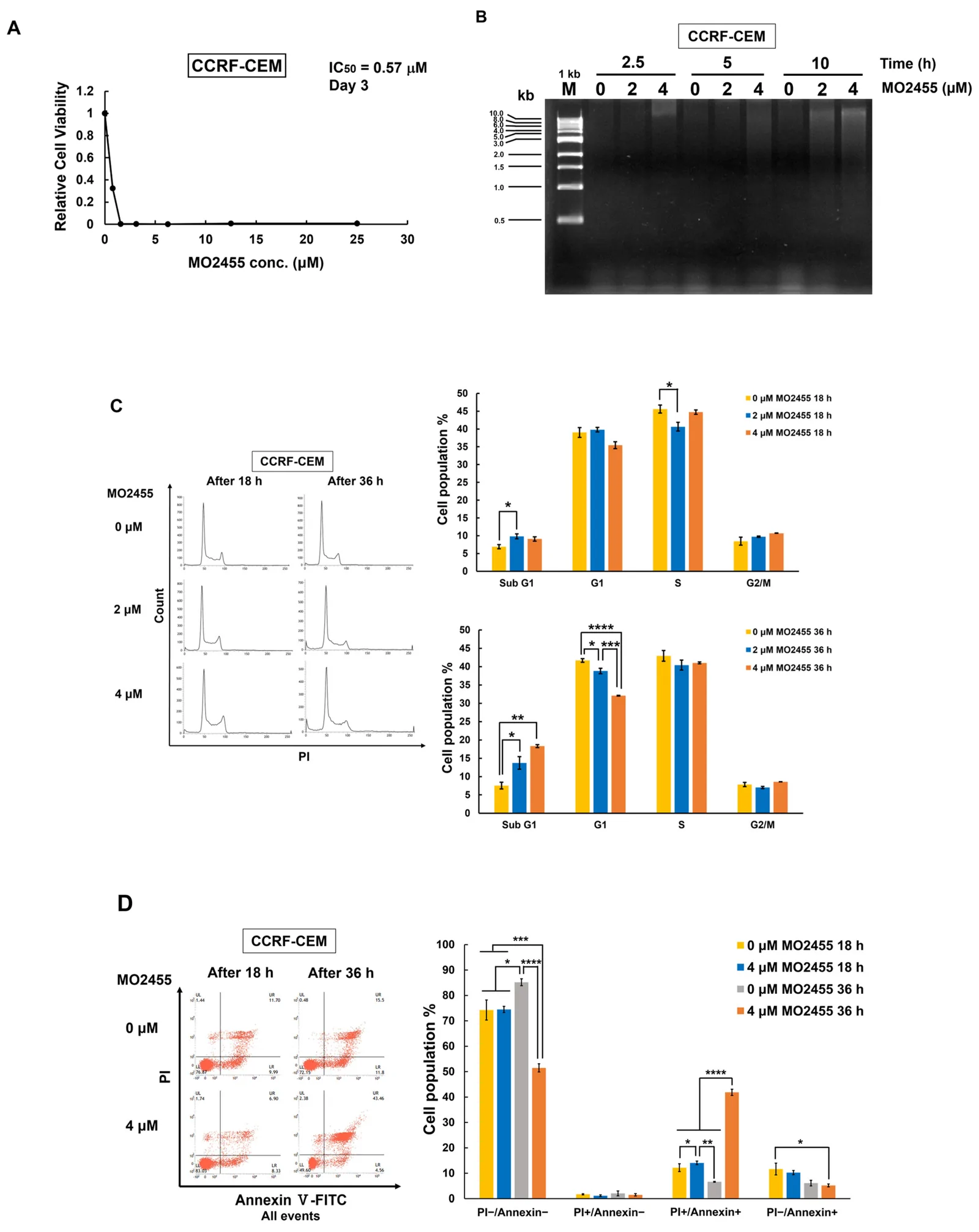
MO2455 induces late apoptosis/necrosis in CCRF-CEM cells. (**A**) Cytotoxicity assessed using the CCK assay on day 3. Cells were treated with different concentrations of MO2455 for an exposure time of 3 days (72 h). Data are presented as the mean ± SE. derived from a representative experiment performed in technical triplicate (n = 3). The IC_50_ value was then calculated. (**B**) Genomic DNA fragmentation analysis in CCRF-CEM cells after MO2455 treatment. Cells were treated with 0, 2, and 4 μM MO2455 for exposure times of 2.5, 5, and 10 h. Data from three independent experiments (n = 3) show reproducible patterns. (**C**) Flow cytometry analysis after MO2455 treatment. Cells were treated with MO2455 at concentrations of 0, 2, and 4 μM for 18 h or 36 h. After PI staining, cell cycle phases were analyzed. (**D**) Cells were treated with MO2455 at concentrations of 0, and 4 μM for 18 h or 36 h and the rate of apoptosis was evaluated using the FITC Annexin V Apoptosis Detection Kit with PI. Data are presented as mean ± SE. Data from three independent experiments (n = 3). Comparisons of all pairs were made using Tukey–Kramer HSD. * *p* < 0.05, ** *p* < 0.001, *** *p* < 0.001, and **** *p* < 0.0001 vs. control.

Cytotoxicity to T-cell leukemia cells was also assessed using CCRF-CEM cells and the CCK assay. Treatment with MO2455 resulted in a concentration-dependent reduction in cell viability, with a calculated IC_50_ value of 0.57 μM, indicating the marked sensitivity of CCRF-CEM cells to MO2455 (Figure 6A). To determine whether the loss of viability was associated with cell death, such as apoptosis, DNA fragmentation was analyzed. As shown in Figure 6B, MO2455-treated CCRF-CEM cells exhibited large-sized (>10 kb) DNA fragmentation similar to THP−1 cells with a more predominant smearing pattern starting from 2.5 h. Treatment with 2 and 4 μM MO2455 for 10 h resulted in more pronounced large-sized DNA fragmentation with smearing. It is thus suggested that in contrast to the typical internucleosomal DNA laddering observed in U937 cells, cell death was not typical of apoptosis in CCRF-CEM cells.

The effect of MO2455 on DNA content and cell cycle was evaluated using flow cytometry analysis of PI-stained cells. MO2455 induced a significant concentration- and time-dependent increase in the sub−G1 population compared to that in the untreated controls (Figure 6C). Accumulation of cells in the sub−G1 population was evident at 2 and 4 μM concentrations, indicating a reduction in DNA amount by fragmentation and cell death. Notably, compared to THP−1 cells, DNA fragmentation level was lower in CCRF-CEM cells at the same MO2455 concentrations. Except for a decrease in the G1 phase in parallel with sub−G1 appearance, no other cell cycle phase changes were observed until 36 h. To further characterize the mode of cell death, Annexin V-FITC/PI staining was performed. MO2455 treatment significantly increased the Annexin+/PI+ population but not the Annexin+/PI− population relative to the untreated controls (Figure 6D). The predominance of Annexin+/PI+ cells with a gradual increase in the sub−G1 population suggests that the cell death process starts rapidly and ends in necrotic cell death mode. These findings demonstrate that MO2455 exerts potent cytotoxic effects in CCRF-CEM cells, leading to rapid DNA fragmentation, accumulation of the sub−G1 population, and augmented Annexin+/PI+ fractions, consistent with necrotic cell death.

### 3.4. Effect of MO2455 on the Viability of Normal Human PBMCs

**Figure 7:**
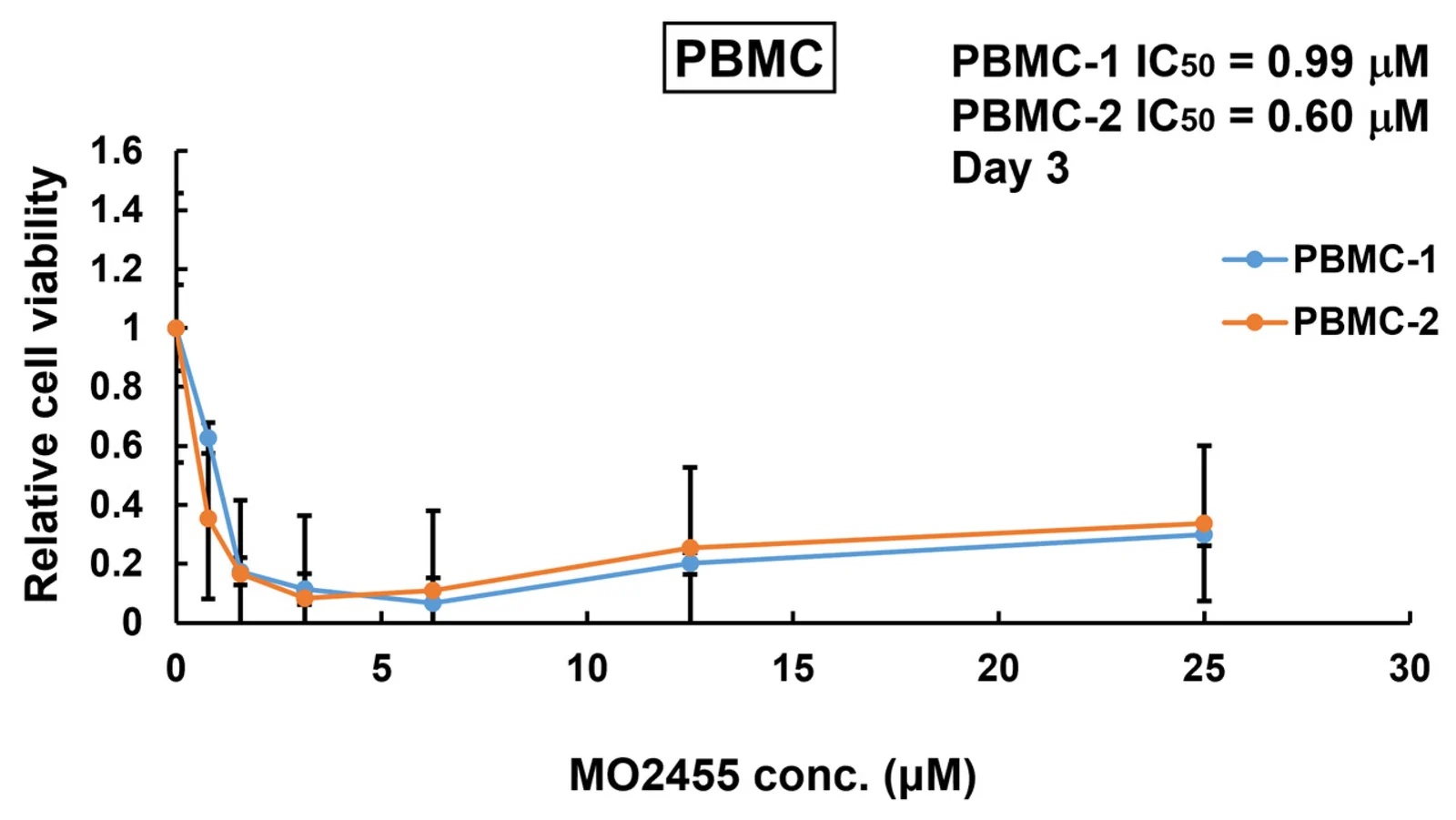
Effect of MO2455 on the viability of normal human PBMCs. The effect of MO2455 on PBMCs isolated from two healthy donors was assessed using the CCK-8 assay. PBMCs were seeded at 2 × 10^4^ cells/well in 96-well plates and treated with different concentrations of MO2455 (up to 25 µM) for 72 h. The peripheral blood obtained from two healthy donors with informed consent was designated as PBMC-1 and PBMC-2, respectively. Data are presented as the mean ± SE of four technical replicates for each donor (n = 4). The apparent IC_50_ values were 0.99 µM for PBMC-1 and 0.60 µM for PBMC-2.

To obtain a preliminary assessment of the effect of MO2455 on normal blood cells, PBMCs isolated from two healthy donors were treated with MO2455, and cell viability was evaluated using the CCK-8 assay (Figure 7).

The IC_50_ of MO2455 in PBMCs from two healthy donors was 0.99 µM, and 0.60 µM for PBMC-1 and PBMC-2, respectively on day 3 after MO2455 treatment. We observed a similar range of toxicity in PBMCs compared to monocytic leukemia U937 cells, whereas cytotoxicity in monocytic leukemia THP−1 and T-cell leukemia CCRF-CEM cells was slightly higher compared to PBMCs.

## 4. Discussion

In this study, we showed the effects of MO2455, a newly synthesized compound. Although MO2455 retains the fragmental trait of imatinib, as shown in Figure 1A,B, MO2455 and imatinib differ in chemical structure by a large percentage. Therefore, we analyzed the biological effect of MO2455 as a new class of chemicals in monocytic leukemia U937, THP−1, and T-cell leukemia CCRF-CEM cells. MO2455 showed cytotoxic effects against all three leukemia cell lines, with IC_50_ values of 0.83 μM, 0.43 μM, and 0.57 μM in U937, THP−1, and CCRF-CEM cells, respectively. Treatment with MO2455 in U937 cells caused early mitochondrial membrane depolarization, significant accumulation of the sub−G1 population, and increased Annexin V positivity, indicating typical apoptotic cell death induction without cell cycle arrest. MO2455 induced γ-H2AX and activated pro-apoptotic *NOXA* and *BBC3* gene upregulation in parallel with apoptosis. THP−1 and CCRF-CEM cells showed necrotic cell death after distinct large-sized DNA fragmentation with smearing but not internucleosomal DNA laddering. This study implies that MO2455 induces cell death in both monocytic and T-cell leukemia through different cell death processes. U937, THP−1, and CCRF-CEM cells commonly harbor p53 mutations or inactivation. U937 cells further possess *PTEN* and *WT1* mutations, *PICALM-MLLT10* fusion mutations, and *PTPN11* activation [30,31,32,33]. THP−1 cells also possess the *NRAS* G12D mutation, *CDKN2A* deletion, and *MTAP* deletion with innate immune and inflammasome activation [30,33,34]. CCRF-CEM cells harbor *STIL–TAL1* fusion, *KRAS* activating mutation, and abnormal TYK2/JAK-STAT signaling [35,36,37]. These different types of mutations may have affected the mode of cell death [34]. At least in U937 and CCRF-CEM cells, cell cycle arrest was not observed after MO2455 treatment, although rapid induction of the sub−G1 population was commonly observed. This suggests that the observed γ-H2AX induction was due to DNA fragmentation into an internucleosomal DNA ladder or large-sized fragmentation in these cells. Unlike U937 cells, THP−1 and CCRF-CEM cells exhibited large-sized fragmentation rather than the characteristic internucleosomal DNA laddering associated with classical apoptosis. This pattern may suggest the involvement of alternative regulated cell death mechanisms, such as parthanatos, which is characterized by PARP1 overactivation and large-sized DNA fragmentation [38,39]. However, the involvement of this pathway was not investigated in the present study, and further studies are required to confirm its role in MO2455-induced cell death.

In this study, U937 cells were selected for detailed mechanistic investigations because they exhibited a clear and comprehensive apoptotic response following MO2455 treatment. Although MO2455 demonstrated potent cytotoxic activity in all three leukemia cell lines, U937 cells showed multiple hallmark features of apoptosis, including mitochondrial membrane depolarization, increased Annexin V positivity, accumulation of the sub−G1 population, internucleosomal DNA fragmentation, γ-H2AX induction, and upregulation of pro-apoptotic genes such as *NOXA* and *BBC3*. These findings indicated that U937 cells provided an appropriate model for further elucidating the molecular events associated with MO2455-induced apoptotic cell death.

The effect of MO2455 on normal PBMCs was examined to obtain preliminary information on its activity toward non-malignant cells. The IC_50_ values were 0.99 µM for PBMC-1 and 0.60 µM for PBMC-2, compared with 0.43, 0.57, and 0.83 µM for THP−1, CCRF-CEM, and U937 cells, respectively. However, these results should be interpreted cautiously because freshly isolated PBMCs are predominantly quiescent and have substantially lower proliferative activity than continuously proliferating leukemia cell lines [40,41]. Since the CCK-8 assay measures cellular metabolic activity, the relatively low absorbance observed in PBMCs may partly reflect their lower metabolic and proliferative activity rather than direct cell death [42]. In addition, the difference observed between the two donors indicates some variability in the response of normal PBMCs to MO2455. Overall, we observed a similar range of toxicity in PBMCs compared to monocytic leukemia U937 cells, whereas cytotoxicity in monocytic leukemia THP−1 and T-cell leukemia CCRF-CEM cells was slightly higher compared to PBMCs. This suggests that at least part of the mechanism of MO2455 in inducing cytotoxicity in leukemia cells and PBMCs may be common. However, because only the CCK-8 assay was performed in PBMCs, these results provide preliminary information on the effect of MO2455 on normal blood cells and do not establish whether the mechanisms underlying the response of PBMCs are similar to or different from those observed in leukemia cells. In the leukemia cell lines, MO2455-induced cell death was associated with DNA fragmentation, loss of mitochondrial membrane potential, accumulation of the sub−G1 population, increased γ-H2AX expression, and changes in cell-death-related genes; however, these mechanistic parameters were not evaluated in PBMCs in the present study. Further studies using complementary viability and cell-death assays are required to confirm its selectivity toward malignant cells. However, we also consider it may be possible to apply MO2455 for therapeutic purposes with specific delivery strategies to cancer cells, which was successful with an antibody-drug conjugate (ADC) to reduce toxicity to normal cells.

One of the limitations of our study is that it was a cell-based study and in vivo evaluation was not conducted. Two monocytic leukemia and one T-cell leukemia cell line were used in this study; therefore, the spectrum of cytotoxicity, including the effect against normal blood cells, should be further examined. Although MO2455 demonstrated potent cytotoxic effects against leukemia cell lines, its selectivity toward malignant cells and potential toxicity to normal hematopoietic cells remain to be fully elucidated. Further investigations using complementary cell death assays and a broader range of normal hematopoietic cell populations will be necessary to determine the therapeutic window and clarify the molecular mechanisms underlying the effects of MO2455 on normal and malignant cells. Future studies comparing MO2455 and imatinib and other structurally related chemicals under the same experimental conditions will help clarify their relative biological activities and mechanisms of action.

Imatinib has been reported to induce mutually exclusive apoptosis and caspase-independent necrosis-like cell death [43]. It has also been reported that imatinib causes caspase-independent cell death, which can be enhanced by inhibiting glycolysis, independent of *Bcr-Abl* or *c-Kit* mutation status [44,45]. Against cardiac progenitor cells, imatinib induced necroptotic cell death at 10 mM [46]. Various imatinib-derived chemicals have been developed, and notably, the derivatives lacking kinase-inhibiting activity also show cell death induction, suggesting that kinase-independent off-target activity for cell death induction is present [20,47]. The current results also support the significance of developing new chemicals possessing some of imatinib’s traits for anti-cancer therapeutics that have multiple ways of cell death induction in cancer cells harboring various types of gene abnormalities or mutations.

For monocytic leukemia, available treatment options remain limited and include conventional chemotherapeutic agents such as cytarabine, anthracyclines, azacytidine, and etoposide, while only a limited number of targeted therapies, including Menin and FLT3 inhibitors, are currently available. Similarly, T-cell leukemia treatment mainly relies on intensive multi-agent chemotherapy involving anthracyclines and antimetabolites such as methotrexate and cytarabine. In this study, the novel imatinib derivative MO2455 demonstrated potent anticancer activity against monocytic and T-cell leukemia cell lines. These findings suggest that MO2455 may represent a promising candidate for further preclinical evaluation, and additional studies are required to determine its therapeutic potential, selectivity, pharmacological properties, and in vivo efficacy.

## Data Availability

The original contributions presented in this study are included in the article/Supplementary Materials. Further inquiries can be directed to the corresponding authors.

## Supplementary Materials

The following supporting information can be downloaded at: https://www.mdpi.com/article/10.3390/cells15191780/s1, File S1: Analytical characterization data of MO2455 by NMR, mass spectrometry and HPLC purity analysis with chromatogram.

## Abbreviations

The following abbreviations are used in this manuscript:

JC-1 dye	5,5, 6,6′-tetrachloro-1,1′, 3,3′-tetraethyl benzimidalyl carbocyanine iodide
