# Supplementary material for: Cell Death Induction by MO2455, a Novel Potential Anti-Cancer Agent in Monocytic and T-Cell Leukemia Cells
Source: Cells. 2026 Sep 30;15(19):1780. doi: 10.3390/cells15191780 (PMC13664501; doi:10.3390/cells15191780)
Supplement: Supplementary file 1 [file cells-15-01780-s001.zip › cells-4454637-file s1.pdf]

Supplementary Materials

Analytical characterization data of MO2455 by NMR, mass spectrometry and HPLC purity analysis with chromatogram is as follows.

5-[[(2,2′-Bithiophen)-5-ylcarbonyl]amino]-*N*-[5-[[4-[4-[(4-ethyl-1-piperazinyl)-carbonyl]phenyl]-2-pyrimidinyl]amino]-2-methylphenyl]- 3-pyridinecarboxamide (MO2455)

1) Purity by HPLC analysis: 97.48%

Sample ID : CR455FFS-9701-25-F
Column : X bridge C-18 (250 x 4.6 mm)
Vial # : 49
Inj. Volume : 4 uL
Tray # : 1
Acquired by : SUBHASH

Data File Name : 01031604 R.lcd
Method File Name : GENERAL B.lcm
Batch File Name :
Data Acquired : 3/1/2016 11:12:32 AM
Data Processed : 3/1/2016 11:43:41 AM
Ref.No: SR/29-02-16/236

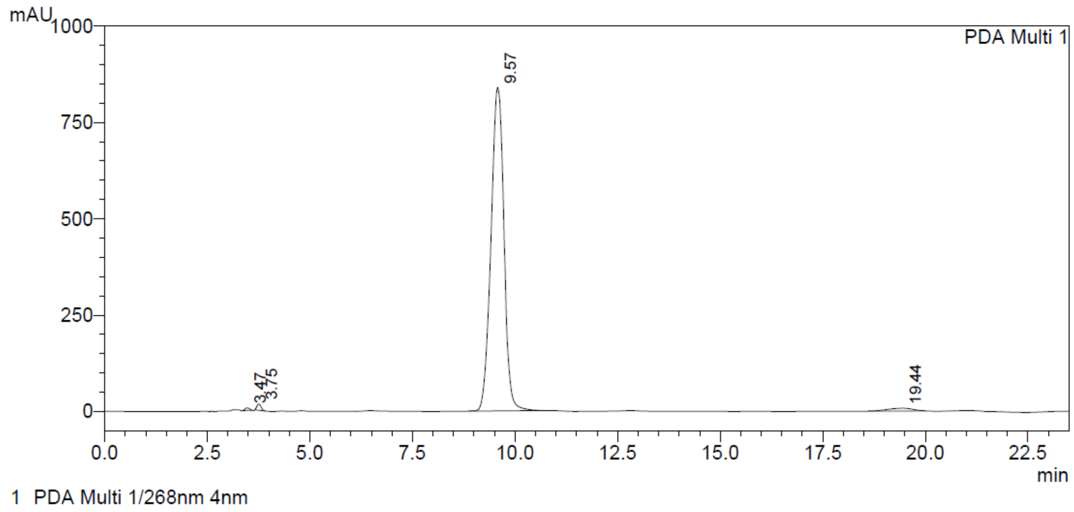

PeakTable

PDA Ch1 268nm 4nm

Peak#	Ret. Time	Area	Area %
1	3.47	50661	0.27
2	3.75	113652	0.60
3	9.57	18411325	97.48
4	19.44	311163	1.65
Total		18886801	100.00

2) NMR analysis: ^{1}H-NMR (300 MHz, DMSO-d_6) δ 1.10 (t, *J* = 7.3 Hz, 3H), 2.23 (s, 3H), 2.88 (brs, 6H), 3.64 (brs, 4H), 7.14 (t, *J* = 3.8 Hz, 1H), 7.23 (d, *J* = 8.0 Hz, 1H), 7.38 (d, *J* = 5.4 Hz, 1H), 7.44 (d, *J* = 3.8 Hz, 1H), 7.47-7.55 (m, 4H), 7.61 (d, *J* = 5.4 Hz, 1H), 8.02 (d, *J* = 4.8 Hz, 1H), 8.13 (s, 1H), 8.20 (d, *J* = 8.0 Hz, 2H), 8.50 (d, *J* = 5.1 Hz, 1H), 8.64 (s, 1H), 8.88 (s, 1H), 8.93 (s, 1H), 9.11 (s, 1H), 10.46 (s, 1H), 10.65 (s, 1H).

3) Mass Spectrometry data: TOF-MS (*m*/*z*): 729.41 $(M+H)^+$.
